# The Phylogenetics and Ecology of the Orthopoxviruses Endemic to North America

**DOI:** 10.1371/journal.pone.0007666

**Published:** 2009-10-29

**Authors:** Ginny L. Emerson, Yu Li, Michael A. Frace, Melissa A. Olsen-Rasmussen, Marina L. Khristova, Dhwani Govil, Scott A. Sammons, Russell L. Regnery, Kevin L. Karem, Inger K. Damon, Darin S. Carroll

**Affiliations:** Coordinating Center for Infectious Diseases, Centers for Disease Control and Prevention (CCID/CDC), Atlanta, Georgia, United States of America; University of Poitiers, France

## Abstract

The data presented herein support the North American orthopoxviruses (NA OPXV) in a sister relationship to all other currently described Orthopoxvirus (OPXV) species. This phylogenetic analysis reaffirms the identification of the NA OPXV as close relatives of “Old World” (Eurasian and African) OPXV and presents high support for deeper nodes within the Chordopoxvirinae family. The natural reservoir host(s) for many of the described OPXV species remains unknown although a clear virus-host association exists between the genus OPXV and several mammalian taxa. The hypothesized host associations and the deep divergence of the OPXV/NA OPXV clades depicted in this study may reflect the divergence patterns of the mammalian faunas of the Old and New World and reflect a more ancient presence of OPXV on what are now the American continents. Genes from the central region of the poxvirus genome are generally more conserved than genes from either end of the linear genome due to functional constraints imposed on viral replication abilities. The relatively slower evolution of these genes may more accurately reflect the deeper history among the poxvirus group, allowing for robust placement of the NA OPXV within Chordopoxvirinae. Sequence data for nine genes were compiled from three NA OPXV strains plus an additional 50 genomes collected from Genbank. The current, gene sequence based phylogenetic analysis reaffirms the identification of the NA OPXV as the nearest relatives of “Old World” OPXV and presents high support for deeper nodes within the Chordopoxvirinae family. Additionally, the substantial genetic distances that separate the currently described NA OPXV species indicate that it is likely that many more undescribed OPXV/NA OPXV species may be circulating among wild animals in North America.

## Introduction

The *Poxviridae* family of viruses is divided into two sub-family groups based on the hosts they infect, vertebrates (Chordopoxvirinae) and invertebrates (Entomopoxvirinae). In general, poxviruses are complex double stranded-DNA viruses with a characteristic brick, or ovoid-shaped virion apparent through electron microscopy. The Chordopoxvirinae are universally distributed and capable of infecting a wide range of avian, reptilian and mammalian species. Some appear to have a limited host range, (*Molluscum contagiosum* and *Variola virus*) while others are found to infect multiple animal species. Many of these poxviruses can cause serious infections in livestock species, including sheep, goats, cattle, crocodiles, caimans, chickens, turkeys and ostriches. Consequently, these pathogens can have devastating effects on livestock production resulting in substantial economic losses. In addition, Chordopoxvirinae species frequently emerge as zoonoses in humans. Sources of human infection include international trade in exotic animals (e.g. *Monkeypox virus*), livestock management (e.g. *Vaccinia virus* and *Orf virus*), and even contact with domestic animals (e.g. *Cowpox virus*). When introduced into non-endemic regions, enzootic chordopoxviruses can threaten native and endemic species previously protected by isolation. *Squirrelpox virus*, presumed to have been imported to the British Isles with the North American grey squirrel (*Sciurus carolinensis*), is thought to have contributed to the rapid decline of red squirrels (*Sciurus vulgaris*) in many areas of the larger islands [1]. Similarly, avipoxviruses have been reported recently as threats to endangered species in the Hawaiian, Canary and Galapagos Islands [2]–[4].

One of the best studied chordopoxvirus genera is *Orthopoxvirus* (OPXV), which includes *Variola virus*, the causative agent of smallpox, and *Vaccinia virus*, the principal source of smallpox vaccine. Additionally, several OPXV species are zoonotic, including *Cowpox virus*, *Monkeypox virus*, and some vaccinia-like virus species. The distribution of most described OPXV species is outside of North America. Those viruses presumed to be endemic to North America currently include only *Raccoonpox virus*, *Skunkpox virus*, and *Volepox virus*. The host range, geographic distribution, and precise taxonomic position of these North American orthopoxviruses (NA OPXV) remain largely undetermined.

The natural reservoir host(s) for many of the described OPXV species remains unknown. However, the ecology and host systems of cowpox viruses have been extensively studied [5]–[7]. *Cowpox virus* species are known to be carried, and hypothesized to be transmitted to humans and domestic animals, by rodent species including bank voles (*Myodes glareolus*) and striped field mice (*Apodemus sylvaticus*). Several other OPXV species are known to be associated with rodents, but the specific nature of these associations remains unknown. *Ectromelia virus* was described from captive colonies of lab mice (*Mus spp*) and some strains are highly lethal to this rodent species, however almost nothing is known regarding its natural distribution [8], [9]. *Monkeypox virus* was initially isolated from captive non-human primates in Denmark [10], and was later associated with human disease [11]. Subsequent ecological studies found OPXV antibodies in a wide variety of African mammalian taxa [12], [13]. The only wild caught animal isolate of *Monkeypox virus* was obtained from a Thomas's rope squirrel (*Funisciurus anerythrus*) collected in Zaire (currently Democratic Republic of Congo) in 1985 [14]. *Taterapox virus* was described in 1975 as an OPXV isolated from a “healthy” naked-soled gerbil (*Tatera kempi*) captured in Benin [15]. An OPXV isolate from northern Brazil was obtained from a rice rat (*Oryzomys sp*.) and found to belong to a larger clade of vaccinia-like viruses which cause disease in cattle and humans throughout the dairy producing regions of Brazil [16], [17]. Smallpox, an exclusively human disease, has been hypothesized to have originated from a rodent-borne zoonotic virus in Africa [18].

Of the NA OPXV, only *Volepox virus* has been definitively associated with rodents, being isolated initially from a scab from a California vole (*Microtus californicus*). OPXV antibodies were found in several other geographically separated populations of this species over a period of two years, suggesting a long term association between the virus and host [19]. An additional isolate was obtained from a Piñon mouse (*Peromyscus truei*) in the same area of the initial isolation site near San Francisco Bay [20]. Typical pock growth on chicken embryonic chorioallantoic membranes (CAM) and hemagglutination-inhibition antibody titers originally revealed the agent to be an OPXV. *Raccoonpox virus* and *Skunkpox virus* were originally each isolated from their namesake mammalian species. A wildlife study conducted across 35,000 acres of Aberdeen Proving Grounds, Maryland, reported the recovery of two *Raccoonpox virus* isolates and a seroconversion rate of 23% in wild raccoons found in the region [21]. No overt disease was noted in the raccoons although the two virus isolates were obtained from “upper respiratory” tissues. OPXV hemaglutination-inhibition antibodies were found in the sera of 22/92 live caught raccoons. A case report from Canada described the occurrence of *Raccoonpox virus* in a domestic cat [22]. “Given the opportunities that free-ranging domestic cats have to encounter infected raccoons, it is somewhat surprising that accidental transmission is not seen more often,” [22]. This report greatly increases the apparent range for *Raccoonpox virus* and raises the possibility of transmission of these poorly characterized viruses to domestic cats, as well as to other domestic or wild animals and perhaps even to humans. A limited infection was reported upon human exposure (needle stick) to a recombinant *Raccoonpox virus* vaccine vector, though natural human infection has yet to be reported [23]. *Skunkpox virus* was isolated from a noticeably ill and presumed (but not) rabid skunk in Colfax, Washington in 1978 and is the only NA OPXV isolated from an overtly ill animal. To further our understanding of the poxviruses circulating in North America, we compare nine genes from across the conserved central region of the poxvirus genome among the eight genera of Chordopoxvirinae. *Skunkpox virus*, *Volepox virus* and *Raccoonpox virus* are included in the analyses to establish the relationship of the North NA OPXV species relative to the currently recognized *Orthopoxvirus* family.

## Results

**Figure 1:**
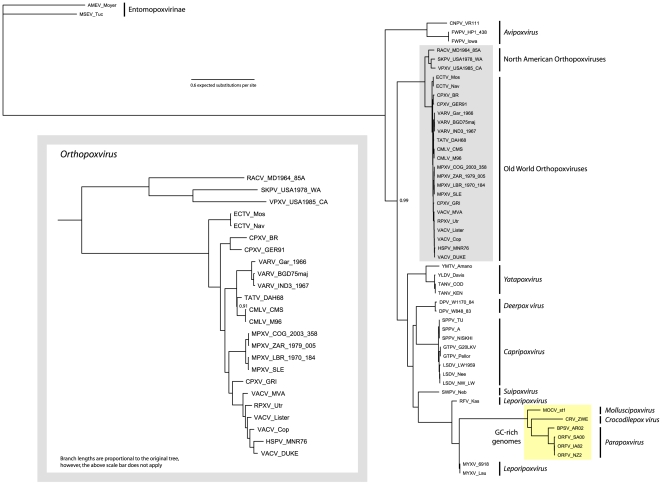
Poxvirus phylogenetic tree inferred from Bayesian analysis of the DNA sequences from 9 conserved genes. Four Metropolis-coupled Markov chain Monte Carlo simulations using the general time reversible (GTR) + γ + I model were run over 5 million generations, with trees and parameters being sampled every 100 generations. The consensus tree was derived from 30,000 of the trees sampled. All but 2 nodes had posterior probabilities of 1.00; these two nodes are labeled in the figure. A close-up of the *Orthopoxvirus* lineage is presented in the inset. GC-rich genomes are indicated by the yellow box.

The phylogenetic tree derived from the selected genes was constructed by Bayesian inference and is shown in Figure 1. Posterior probabilities were consistently high (≥.90) for all depicted clades on the phylogram, only (the two) values less than 1.00 are shown. These values indicate that there is very strong support for the relationships depicted. The avipoxviruses (CNPV and FWPV) are sister to a clade containing all other chordopoxvirus taxa. Among taxa of the genus OPXV, a monophyletic European and African OPXV clade is depicted and is joined by the species of the NA OPXV. Within the NA OPXV clade, *Raccoonpox virus* (RACV) forms a monophyletic group with a *Skunkpox virus*/*Volepox virus* (SKPV, VPXV) sister grouping. The relative branch lengths between the NA OPXV and leading to other orthopoxviruses illustrates the considerable distance between the two clades (NA OPXV and other OPXV) and that the NA OPXV have much greater distances between species than is seen among the recognized African and Eurasian OPXV species. Outside of the OPXV clade a monophyletic *Yatapoxvirus* clade is sister to a larger non-OPXV clade. This non-OPXV/non-*Yatapoxvirus* clade consists of two clades. *Deerpox virus* (DPV) isolates are sister to a *Capripoxvirus* clade which is consistent with previous analyses [24]. The clade of capripoxviruses consists of *Sheeppox virus* (SPPV) sister to a monophyletic clade including *Lumpy skin disease virus* (LSDV) and *Goatpox virus* (GTPV). The second clade consists of *Swinepox virus* (SWPV), *Rabbit fibroma virus* (RFV), *Myxoma virus* (MYXV), *Molluscum contagiosum virus* (MOCV), *Crocodilepox virus* (CRV), *Bovine papular stomatitis virus* (BPSV) and *Orf virus* (ORFV) which form a ladder configuration beginning with LSDV as the first to split from the others and ending with BPSV and ORFV in a sister grouping.

Pairwise genetic distances and branch lengths between NA OPXV species and their nearest Old World cousin, ECTV_Mos (*Ectromelia virus*) are presented in Table 1. The Old World virus species most distant from ECTV_Mos is VAR_IND3_1967 (*Variola virus*) (patristic distance  = 0.033874). Comparatively, the average patristic difference between ECTV_Mos and NA OPXV species (0.160133) is nearly five times the distance between ECTV_Mos and VARV_IND3_1967. Similarly, the two closest NA OPXV species, *Volepox* (VPXV) and *Skunkpox virus* (SKPV), lie at a patristic distance (0.070158) twice that of ECTV_Mos and VARV_IND3_1967. The results of the Shimodaira-Hasegawa test which analyzed the log likelihood scores of alternative tree topologies (with each NA OPXV constrained into the Old World OPXV clade) indicate that the original tree, with a monophyletic NA OPXV, has a significantly (P<.05) better topology.

**Table 1:** Pairwise genetic distances* (lower) and patristic distances (upper).

	RACV_MD 1964_85A	SKPV_USA 1978_WA	VPXV_USA 1985_CA	ECTV_Mos
RACV_MD 1964_85A	-	0.1083	0.1129	0.1658
SKPV_USA 1978_WA	0.101318	-	0.0732	0.1729
VPXV_USA 1985_CA	0.105718	0.070158	-	0.1775
ECTV_Mos	0.161211	0.158118	0.16107	-

*Distance measure  =  general time-reversible model; proportion of sites assumed to be invariable  = 0.0727; identical sites removed proportionally to base frequencies estimated from all sites (distances represent mean number of substitutions over all sites); rates (for variable sites) assumed to follow gamma distribution with shape parameter  = 0.976.

The genomes of *Molluscum contagiosum* (MOCV_st1), *Crocodilepox virus* (CRV_ZWE), and the parapoxviruses (BPSV_AR02, ORFV_AS00, ORFV_IA82, ORFV_NZ2) are unusually GC-rich compared to the rest of the *Poxviridae* [25]. MOCV_st1 contains 64% GC while CRV_ZWE has 62% and parapoxviruses average 64% [26]–[28]. The members of these three species share the highest numbers for GC content among poxviruses. The only other poxvirus genomes that come close to this level of GC content are those of the genus *Leporipoxvirus* (43.6% and 39.5% for MYXV and RFV respectively) [29], [30]. This similarity in nucleotide composition is a likely explanation for the loose grouping of these species in the phylogenetic tree. *Molluscum contagiosum virus* and *Orf virus* have been presented in a similar topology in previous studies [25], [27], [31]. To overcome this confounding factor, we ran an additional analysis using the same DNA matrix but excluding species with high GC content (2,655,100 iterations, sampling every 1000 generations, standard deviation of split frequencies 0.000346, burnin 6,000). The loss of the GC-rich genomes shortened all branch lengths; however, the resultant topology was identical to the previous analysis (Figure 1) in the placement of the NA OPXV as sister to the remaining orthopoxviruses. Removing the GC rich genomes resulted in a monophyletic *Leporipoxvirus* clade and moved *Suipoxvirus* from a sister position with *Leporipoxvirus* to a sister relationship with *Deerpox virus*. The large non-*Orthopoxvirus* clade is still formed by *Yatapoxvirus*, *Leporipoxvirus*, *Deerpox virus* and *Capripoxvirus* added in a stepwise manner from the deepest node. The relationship between *Suipoxvirus*, *Deerpox virus* and *Capripoxvirus* has been shown to be close in previous work [24] and a similar, non-*Orthopoxvirus* clade structure is presented in Bratke and McLysaght [32].

## Discussion

### OPXV Host associations

Members of the OPXV genus infect several mammalian taxa as both enzootic and zoonotic pathogens, and in at least some cases are present asymptomatically. In terms of isolates obtained from wild animals, rodents are the most often associated mammalian taxon regarding the isolation of the currently described OPXV species. *Volepox virus*, *Cowpox virus*, vaccinia-like viruses, and *Taterapox virus* are all believed to infect rodent species with little obvious disease in the host although, with the exception of *Cowpox virus*, only limited data are available [5], [15], [19], [33]. Within the genus OPXV, the host-virus relationship resulting in minimal or asymptomatic infection (in rodents) is distributed widely throughout the OPXV viral phylogeny. This pattern along with the high degree of genetic similarity between OPXV species suggests that rodents could represent the primary ecological reservoir of the members of this genus. Currently there are only three enzootic (*Camelpox virus* and *Taterapox virus*), or zoonotic (*Monkeypox virus*) OPXV species described as endemic to the African continent, and the genomic divergence between these viruses is relatively slight. Most of the African OPXV ecological surveillance studies have used serological assays that cross react between members of the OPXV genus and are not able to distinguish between exposures to different OPXV species. This, along with the low number of ecology studies relative to the size and ecological diversity of the African Continent, make it likely that there are several more African OPXV species that have not yet been described.

The topology and the relatively large patristic distances between NA OPXV and the other OPXV species indicate that the NA OPXV species are the most divergent members of OPXV currently described and are some of the least studied. All of these are mammal-associated and two of the three (*Raccoonpox virus* and *Volepox virus*) have not been linked to any obviously pathogenic disease in animals or humans. Ecological studies of these two viruses indicate widespread and possibly long-term associations between the viruses and the mammal species from which they were isolated [19], [21]. Although *Skunkpox virus* was isolated from a sick animal, it is not clear if the virus was the cause of the illness. The recent occurrence of *Raccoonpox virus* in a domestic cat in Canada illustrates the possibility of cross-species transmission of these viruses. Reports of *Cowpox virus* in domestic cats and humans have been occurring with increased frequency in Europe [34], [35], but the range of this/these virus species has not been reported to include the Americas. The rise in Old World human *Cowpox virus* infections may be attributable to the decrease in number of persons vaccinated for smallpox and the loss of cross-protection against other poxvirus species. These conditions also exist in North America and could lead to an increase in the transmission of NA OPXV to humans from the reservoir species or domestic animals.

### Classification of OPXV species

It has been suggested that cowpox viruses represent the ancestral state of OPXV species in that they possess every gene present in every other OPXV [36], [37]. In this sense, by retaining perhaps all of the original genes of the common ancestor of OPXV, cowpox viruses likely resemble the ancestor of this clade in gene composition. However, there is no evidence that the OPXV are direct descendants of any known extant OPXV species (i.e. cowpox viruses have evolved from the common ancestor of OPXV just as the other species have). The general evolutionary trend within the OPXV genus is the loss of genomic material [38]. The phylogeny produced in the current analyses was generated from nine coding regions present in all members of the genus *Orthopoxvirus* as well as the members of the *Poxviridae* and would not be influenced by gene loss. *Ectromelia virus* (ECTV_Nav, ECTV_Mos), also one of the larger genomes, is consistently sister to the remaining non-NA OPXV *Orthopoxvirus* group in this and other analyses [39], [41]. The short length of the ECTV branch suggests that these ECTV species have accumulated fewer nucleotide changes, with respect to the other OPXV species, since the split from the most recent OPXV common ancestor. In addition, the initial divergence within the NA OPXV appears to have occurred much earlier than the apparently more recent and/or rapid radiation of known Old World OPXV. However, the inclusion of additional species not currently described from both Old and New World OPXV clades would almost certainly give better phylogenetic resolution in future analyses.

As seen in previous studies [37], *Cowpox virus* isolates (CPXV_BR, CPXV_Ger-91 and CPXV_GRI) do not form a monophyletic clade. CPXV_GRI is most closely related to the vaccinia virus group, while CPXV_BR diverges from a deeper node on the tree. The phylogenetic and taxonomic incongruence of “cowpox virus”, despite some similar virological properties including chorioallantoic pock morphology, is likely the result of multiple OPXV species which cause a rash illness in cows. The traditional common names assigned to poxviruses are often confusing, as the disease presentation is superficially similar for many of the pathogenic species. Cows are susceptible to both *Vaccinia virus* and the multiple *Cowpox virus* clades. Lineages of these similar viruses may explain some of the historic confusion regarding cowpox, horsepox and vaccinia with regard to the condition and the causative viral agents [40]. These phylogenetic analyses indicate that a taxonomic review of “cowpox virus” is warranted. Additionally, this phylogeny depicts a recent common ancestor shared between CPXV_GRI and the vaccinia clade, which may provide clues into the origins of *Vaccinia virus*. All of the vaccine and sylvan (from wild animals) isolates form a monophyletic clade which justifies the classification of all members of this clade under the species *Vaccinia virus*.

The data presented herein support a monophyletic NA OPXV clade in a sister relationship to all other currently described OPXV species. The genus OPXV has been defined traditionally by some combination of: the presence of cross-neutralizing antibodies within members of the genus, genomic similarities, biologic phenotypes, similar immunodiffusion patterns, and hemagglutination inhibition activity. Hemagglutination-inhibition antibody titers were reported at 640 and 1280 (reciprocals of the highest inhibiting dilution) for three samples (3.3%) of raccoon sera against *Vaccinia virus*, while initial studies demonstrated little to no hemagglutination inhibition (0 to 160) between vole sera and *Vaccinia virus* or *Raccoonpox virus* [19], [21]. However, immunodiffusion and complement fixation test results suggest that the NA OPXV are most closely allied to the traditional members (*Vaccinia virus*, *Variola virus* and *Cowpox virus*) of the OPXV. Further studies are needed to clarify the classification of the group based on biologic criteria.

The current, gene sequence based phylogenetic analysis reaffirms the identification of the NA OPXV as the nearest relatives of “Old World” OPXV and presents high support for deeper nodes within the Chordopoxvirinae family. Two of the three NA OPXV species are known only from isolates obtained from carnivores. If these Carnivore species represent the natural host(s), then either these viruses have a very taxonomically broad host range or the origins of the genus OPXV could be much more ancient than previously thought, dating back to the common ancestor of the Rodentia and Carnivora. Given the relatively high genetic divergence within the NA OPXV clade it is conceivable that the hosts could be as taxonomically distant as rodents and carnivorans. However, it is also readily conceivable that the carnivoran species could have been infected by contact with rodents as with cowpox viruses in the Old World. Future ecological investigations are needed to refine the understanding of the ecological parameters which result in the maintenance of these NA OPXV viruses in the environment. Two scenarios could explain the observed relatively high genetic diversity within the NA OPXV. First, if one suggests that these are the only OPXV viruses currently native to North America, one must posit that there have been substantial virus extinction events during the course of NA OPXV evolution leaving behind a very small number of highly divergent relic species. Alternatively,the substantial genetic distances that separate the currently described NA OPXV species could indicate that many more undescribed extant OPXV/NA OPXV species may be circulating among wild animals in North America.

## Methods

### Virus Isolates/Strains and Sequencing

All analyzed strains represent low passage (1–2 passages) isolates that are as close to the “wild” type viruses as possible. The isolates chosen for analysis were, when possible, from the original specimen described for the species. Genes from the central region of the poxvirus genome are generally more conserved than genes from either end of the linear genome due to functional constraints imposed on viral replication abilities. The relatively slower evolution of these genes may more accurately reflect the deeper history among the poxvirus group, allowing for robust placement of the NA OPXV within Chordopoxvirinae. Sequencing of three NA OPXV genomes was carried out using standard protocols of the Roche GS-20 pyrosequencing platform (Roche Applied Science, Indianpolis, IN) and confirmatory Sanger sequencing. Sequence data for nine genes were compiled from these strains plus an additional 50 genomes collected from Genbank. A list of strains, isolates, and sources is available in Table 2.

**Table 2:** 

**Entomopoxvirinae**			
Code	**Genus**	Strain	Accession No.
	Species		
	***Betaentomopoxvirus***		
AMEV	*Amsacta moorei entomopoxvirus 'L'*	Moyer	NC_002520
	**Undefined**		
MSEV	*Melanoplus sanguinipes entomopoxvirus*	Tucson	NC_001993
**Chordopoxvirinae**			
Code	**Genus**	Strain	Accession No.
	Species		
	***Orthopoxvirus***		
CMLV	*Camelpox virus*	CMS	AY009089
		M96	NC_003391
VARV	*Variola virus*	Bangladesh 1975	L22579
		India 1967	NC_001611
		Garcia 1966	Y16780
TATV	*Taterapox virus*	Dahomey 1968	NC_008291
CPXV	*Cowpox virus*	Brighton Red	NC_003663
		GRI-90	X94355
		Germany 91-3	DQ437593
VACV	*Vaccinia virus*	Lister	AY678276
		Duke	DQ439815
		Copenhagen	M35027
		Modified vaccinia Ankara	U94848
HSPV	(*Horsepox virus*)*	MNR-76	DQ792504
RPXV	(*Rabbitpox virus*)*	Utrecht	AY484669
MPXV	*Monkeypox virus*	Zaire 1979	DQ011155
		Sierra Leone	AY741551
		Liberia 1970	DQ011156
		Congo 2003	DQ011154
ECTV	*Ectromelia*	Moscow	NC_004105
		Naval	None
RACV	*Raccoonpox virus*	MD1964-85A	FJ807746-54
SKPV	*Skunkpox virus*	USA1978-WA	FJ807755-63
VPXV	*Volepox virus*	USA1985-CA	FJ807737-45
	***Parapoxvirus***		
ORFV	*Orf virus*	NZ2	DQ184476
		IA82	AY386263
		SA00	NC_005336
BPSV	*Bovine papular stomatitis virus*	BV-AR02	NC_005337
	***Molluscipoxvirus***		
MOCV	*Molluscum contagiosum virus*	Subtype 1	NC_001731
	***Leporipoxvirus***		
MYXV	*Myxoma virus*	Lausanne	NC_001132
		6918	EU552530
RFV	*Shope fibroma virus*	Kasza	NC_001266
	***Suipoxvirus***		
SWPV	*Swinepox virus*	Nebraska 17077-99	NC_003389
	***Capripoxvirus***		
GTPV	*Goatpox virus*	G20-LKV	AY077836
		Pellor	NC_004003
LSDV	*Lumpy skin disease virus*	Neethling 2490	NC_003027
		Neethling vaccine LW 1959	AF409138
		Neethling Warmbaths LW	AF409137
SPPV	*Sheeppox virus*	Strain A	AY077833
		NISKHI	AY077834
		TU-V02127	NC_004002
	***Yatapoxvirus***		
TANV	*Tanapox virus*	TPV-RoC	EF420157
		Kenya	NC_009888
		YLDV-Davis	NC_00264
YLDV	*Yaba monkey tumor virus*	Amano	NC_005179
	***Avipoxvirus***		
CNPV	*Canarypox virus*	ATCC VR111	NC_005309
FWPV	*Fowlpox virus*	HP1-438 Munich	AJ581527
		FCV Iowa	NC_002188
	**Undefined**		
CRV	*Crocodilepox virus*	Zimbabwe	NC_008030
DPV	*Deerpox virus*	W-1170-84	AY689437
		W-848-83	NC_006966

*also a strain of *vaccinia virus*, though commonly referred to by the listed name.

### Alignment

Nine coding sequences were selected from the central region of the genome: A7L, early transcription factor/VETF large subunit (82 kDa); A10L, major core protein; A24R, RNA polymerase 132; D1R, messenger RNA capping enzyme, large subunit; D5R, DNA independent NTPase (DNA replication); E6R, hypothetical protein; E9L, DNA polymerase; H4L, RNA polymerase associated protein; J6R, RNA polymerase 147. Gene designations refer to the VACV-COP genome. Seven of these coding regions make up a subset of the ‘core families’ used to estimate a poxvirus phylogeny by Bratke and McLysaght [32]. By necessity, all nine genes are included in the 49 genes completely conserved among poxviruses [41]. Multiple sequence alignment of amino acids was performed using the ProbCons [42] server available from the Max Plank Institute for Developmental Biology (http://toolkit.tuebingen.mpg.de/probcons). The RevTrans 1.4 Server (http://www.cbs.dtu.dk/services/RevTrans/) was then used to translate the amino acid alignment back into the original DNA sequence [43].

### Phylogenetic Analysis

Phylogenetic analysis was carried out on the concatenated DNA alignments using Bayesian inference as executed by MrBayes using four “chains” run over five million generations [44], [45]. Modeltest was used to identify the most appropriate model of evolution for both the entire dataset and for each gene individually. The identified program settings for all partitions, under the Akaike Information Criteria, included six character states (General Time Reversible model), a proportion of invariable sites, and a gamma distribution of rate variation across sites (GTR+I+G). A second Bayesian analysis was conducted wherein genes were partitioned. Parameters of each partition were allowed to vary independently and each partition was allowed to evolve under a different rate. The resulting tree shared the same topology as the tree recovered in the initial analysis. In addition, an incongruence length difference (a.k.a. partition homogeneity) test was performed in which partitions were defined by gene. The results (P = 0.01) indicated that the phylogenetic signals from the genes were not significantly incongruent to justify separate analyses. Because the placement of NA OPXV among poxviruses has yet to be tested by a multigene phylogenetic analysis, we included representatives from ten Chordopoxvirinae genera and two Entomopoxvirinae species to serve as outgroup taxa. PAUP* [46] was used to calculate genetic distances (based on the maximum likelihood parameters used in MrBayes) in order to compare these and the patristic distances (based on tree branch lengths) between taxa.

In order to examine the validity of the monophyletic NA OPXV we constructed 3 constrained trees in which each of the NA OPXV species were separately excluded from the monophyletic cluster and forced into monophyly with the “Old World” OPXV (see Figure 1). The Shimodaira-Hasegawa test (implemented in PAUP* with 1000 replicates) was then used to compare the resultant log likelihood scores of each constrained tree to the original tree.
